# HEXA-018, a Novel Inducer of Autophagy, Rescues TDP-43 Toxicity in Neuronal Cells

**DOI:** 10.3389/fphar.2021.747975

**Published:** 2021-12-02

**Authors:** Shinrye Lee, Myungjin Jo, Hye Eun Lee, Yu-Mi Jeon, Seyeon Kim, Younghwi Kwon, Junghwa Woo, Shin Han, Ji Young Mun, Hyung-Jun Kim

**Affiliations:** ^1^ Dementia Research Group, Korea Brain Research Institute (KBRI), Daegu, South Korea; ^2^ Neural Circuit Research Group, Korea Brain Research Institute (KBRI), Daegu, South Korea; ^3^ Department of Brain and Cognitive Sciences, DGIST, Daegu, South Korea; ^4^ Hexa Pharmatec, Ansan-si, South Korea

**Keywords:** catechol, autophagy, mitochondrial dysfunction, TDP-43, ALS

## Abstract

The autophagy-lysosomal pathway is an essential cellular mechanism that degrades aggregated proteins and damaged cellular components to maintain cellular homeostasis. Here, we identified HEXA-018, a novel compound containing a catechol derivative structure, as a novel inducer of autophagy. HEXA-018 increased the LC3-I/II ratio, which indicates activation of autophagy. Consistent with this result, HEXA-018 effectively increased the numbers of autophagosomes and autolysosomes in neuronal cells. We also found that the activation of autophagy by HEXA-018 is mediated by the AMPK-ULK1 pathway in an mTOR-independent manner. We further showed that ubiquitin proteasome system impairment- or oxidative stress-induced neurotoxicity was significantly reduced by HEXA-018 treatment. Moreover, oxidative stress-induced mitochondrial dysfunction was strongly ameliorated by HEXA-018 treatment. In addition, we investigated the efficacy of HEXA-018 in models of TDP-43 proteinopathy. HEXA-018 treatment mitigated TDP-43 toxicity in cultured neuronal cell lines and *Drosophila*. Our data indicate that HEXA-018 could be a new drug candidate for TDP-43-associated neurodegenerative diseases.

## 1 Introduction

The autophagy-lysosomal pathway (ALP) is an evolutionarily conserved catabolic mechanism that involves the degradation of unnecessary or abnormal proteins/organelles (Lee, 2012). The postmitotic and long-lived nature of neurons makes them vulnerable to proteotoxic stress induced by the accumulation of misfolded proteins or damaged organelles (Son et al., 2012). Thus, maintaining efficient ALP function is essential for neuronal survival, and dysfunction of the ALP is one of the common features of neurodegenerative diseases (Son et al., 2012).

TDP-43 is part of an evolutionarily conserved family of heterogeneous nuclear ribonucleoproteins that modulate multiple steps of RNA metabolic processes (Jo et al., 2020). Therefore, TDP-43 is mainly localized in the nucleus but also shuttles between the nucleus and the cytoplasm to perform various cellular functions. However, under pathological conditions, cytoplasmic transfer of TDP-43 increases, and mislocalized TDP-43 accumulates in the cytoplasm, which could contribute to neuronal dysfunction and toxicity (Van Deerlin et al., 2008). Cytoplasmic aggregation of TDP-43 in affected neurons is a pathological hallmark of many neurodegenerative diseases, including amyotrophic lateral sclerosis (ALS), frontotemporal dementia (FTD), Alzheimer’s disease (AD), and limbic predominant age-related TDP-43 encephalopathy (LATE) (Huang et al., 2020).

Previous studies have shown that gain of function or overexpression of TDP-43 in neuronal cells is sufficient to cause neurodegeneration. Mislocalized and accumulated TDP-43 induces mitochondrial dysfunction and reactive oxidative species (ROS) production (Wang et al., 2019). Furthermore, oxidative stress exacerbates the cytotoxicity of TDP-43 (Prasad et al., 2019). Another mechanism of TDP-43-induced neurotoxicity is impairment of the ubiquitin proteasome system (UPS). The UPS is one of the major intracellular protein quality control systems, and it is a critical regulator of misfolded and aggregation-prone proteins, which have been found to accumulate in neurodegenerative diseases. Recent studies have shown that UPS impairment is implicated in the neurotoxicity of TDP-43 in mammalian cell models and *Drosophila* (Lee et al., 2020a; Lee et al., 2020b). In addition, TDP-43 toxicity is significantly suppressed by ALP activation (Barmada et al., 2014; Lee et al., 2020a). Moreover, some genes associated with TDP-43 proteinopathy, such as *SQSTM1*, valosin-containing protein (*VCP*), optineurin (*OPTN*), and TANK binding kinase 1 (*TBK1*), are closely linked to the ALP (de Boer et al., 2020). Therefore, modulation of the ALP could be a potential therapeutic approach for TDP-43 proteinopathy.

**FIGURE 1:**
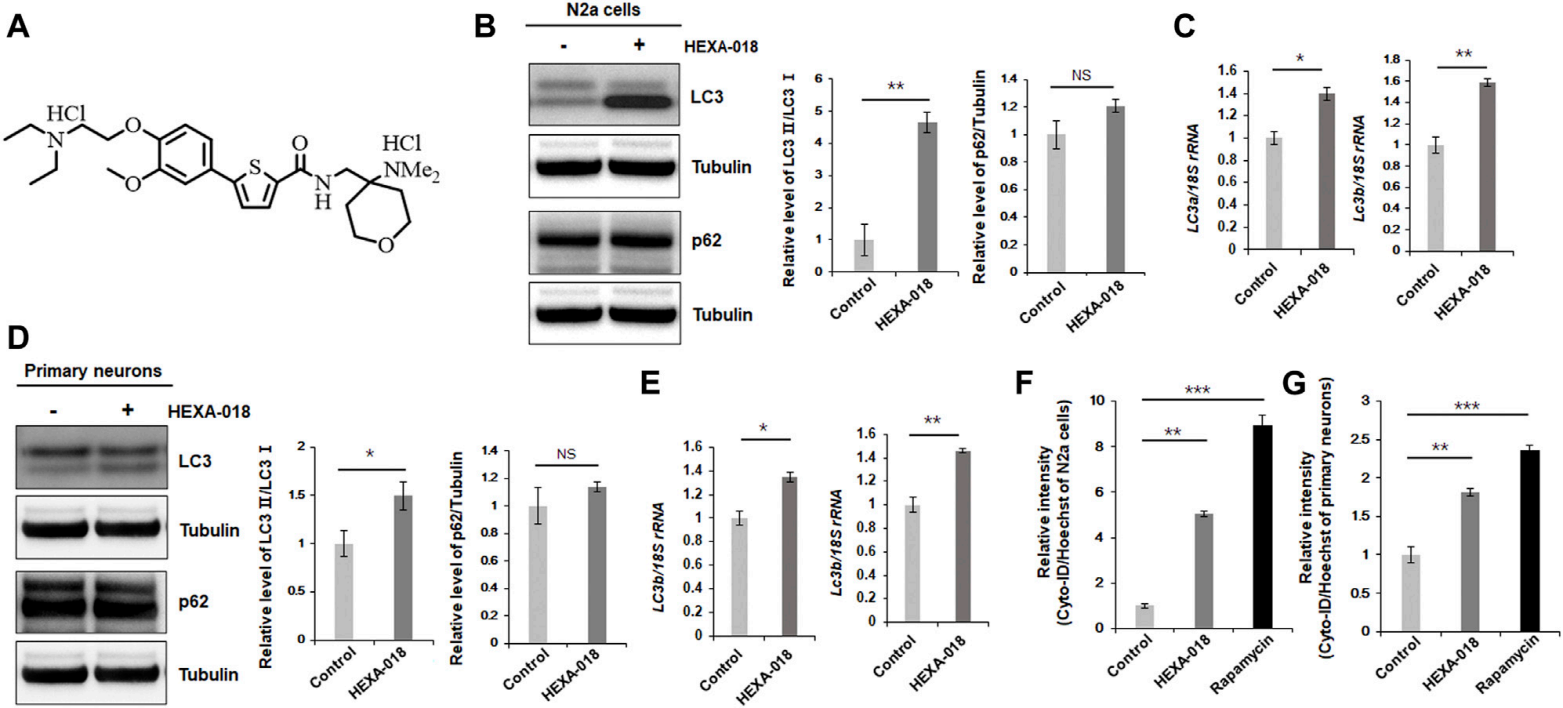
HEXA-018 induces autophagy in neuronal cells. **(A)** Chemical structure of HEXA-018. **(B,C)** N2a cells were treated with HEXA-018 (5 µM) for 24 h. **(B)** Western blot analysis was performed to determine the amount of LC3-I/II or p62 protein. HEXA-018 significantly increased the LC3-II levels in N2a cells but did not affect total p62 protein levels. Quantification of the immunoblots was performed from 3 independent experiments. **p < 0.005; *n. s.*, not significant (unpaired Student’s *t*-test). **(C)** RT-PCR for *lc3a* and *lc3b* mRNA expression in HEXA-018-treated N2a cells. The quantification of *lc3a* and *lc3b* mRNA transcript levels is presented as the mean ± SEM from 3 independent real-time RT-PCR experiments. *18S rRNA* was used for normalization. *p < 0.05; **p < 0.005 (unpaired Student’s *t*-test). **(D,E)** Primary neurons were treated with HEXA-018 (0.5 µM) for 24 h. **(D)** Western blot analysis was performed to determine LC3-I/II or p62 protein expression. Quantification of the immunoblots was performed from 3 independent experiments. *p < 0.05; *n. s.,* not significant (unpaired Student’s *t*-test). **(E)** RT-PCR for *lc3a* and *lc3b* mRNA expression in HEXA-018-treated primary neurons. The quantification of *lc3a* and *lc3b* mRNA transcription levels are presented as the mean ± SEM from 3 independent real-time RT-PCR experiments. *18S rRNA* was used for normalization. *p < 0.05; **p < 0.005 (unpaired Student’s *t*-test). **(F,G)** N2a cells **(F)** and primary neurons **(G)** were treated with HEXA-018 (5 µM/0.5 µM) for 24 h. Rapamycin (200 nM) was used as a positive control. Cyto-ID fluorescence analysis was performed thereafter. The level of Cyto-ID fluorescence was significantly increased in the HEXA-018-treated cells, which indicates autophagic activation. The fluorescence signal intensity of Cyto-ID was normalized to that of Hoechst 33342. Data are presented as the mean ± SD from 3 independent experiments. **p < 0.005; ***p < 0.001 (one-way ANOVA with Tukey’s multiple comparison test).

N-((4-(Dimethylamino)tetrahydro-2H-pyran-4-yl)methyl)-5-(4-(2-diethylamino)ethoxy-3-methoxyphenyl)thiophene-2-carboxamide hydrochloride (also called HEXA-018, Figure 1A) is a newly developed inducer of autophagy, but its effect in neuronal cells has not been studied (Han and Lee, 2020). In this study, we found that HEXA-018, a novel compound containing a catechol derivative structure, activated the autophagic pathway via an mTOR-independent pathway and mitigated neuronal toxicity induced by oxidative stress and ubiquitin proteasome system (UPS) impairment. Both oxidative stress and UPS impairment are major pathological features of TDP-43 proteinopathy. Moreover, HEXA-018 treatment reduced TDP-43-induced neurotoxicity in cells and *Drosophila*. Therefore, we expect that the novel catechol derivative compound HEXA-018 might be a drug candidate for neurodegenerative diseases associated with TDP-43 accumulation.

## 2 Materials and Methods

### 2.1 Reagents and Antibodies

The following reagents were purchased from the indicated providers: dimethyl sulfoxide (DMSO; Sigma, D8418), MG132 (Calbiochem/Merck-Millipore, 474791), rotenone (Sigma, R8875), rapamycin (InvivoGen, tlrl-rap), ULK1 inhibitor (MRT68921; Selleckchem, S7949), and mifepristone (RU-486; Sigma, M8046). We also received HEXA-018 from Hexa Pharmatec., which is not commercially available (Patent number, WO 2020/017878 A1).

The following antibodies were used for immunoblotting: GFP (Clontech, 632380), p62 (Sigma, P0067), LC3 (MBL, PM036), phospho-mTOR Ser2448 (Cell Signaling, 5536), phospho-mTOR Ser2481 (Cell Signaling, 2974), mTOR (Cell Signaling, 2983), phospho-ULK1 Ser757 (Cell Signaling, 6888), ULK1 (Abcam, ab128859), phospho-AMPKα1 Thr183 + AMPKα2 Thr172 (GeneTex, GTX130429), AMPKα1/α2 (GeneTex, GTX50863), Ref2(P) (Abcam, ab178440), HRP-conjugated anti-alpha-tubulin (Cell Signaling, 9099), HRP-conjugated rabbit IgG (Santa Cruz Biotechnology, sc-2004), and HRP-conjugated mouse IgG (Santa Cruz Biotechnology, sc-2005).

### 2.2 Cell Lines

The Neuro-2a (ATCC, N2a) mouse neuroblastoma cell line was maintained in Dulbecco’s modified Eagle’s medium (DMEM; Gibco, 11995-065) supplemented with 10% heat-inactivated fetal bovine serum (FBS; Gibco, 16000-044) and 50 μg/ml penicillin-streptomycin (Gibco, 15140-122). Cells were grown at 37°C in a humidified atmosphere containing 5% CO_2_. All experiments were performed using cells from passages 5 to 15.

### 2.3 Mouse Cortical Neuron Cultures

Primary cultures of cerebral cortical neurons were prepared from 16-days embryonic mice as described previously (Enokido et al., 1992; Araki et al., 2000). Briefly, mouse embryos were decapitated, and the brains were rapidly removed and placed in a culture dish containing HBSS (Gibco, 14170-112). Cortices were isolated, transferred to a conical tube and washed twice in HBSS. Cortical tissues were enzymatically digested by 20 units/ml papain (Worthington Biochemical Corporation, LK003150) and 0.005% DNase I for 30 min at 37°C. The tissues were mechanically dissociated by gently pipetting up and down. The cortical cells were centrifuged at 800 × *g* for 10 min at room temperature. Then, the dissociated cells were seeded on plates coated with poly-D-lysine (Sigma-Aldrich, P7405) in neurobasal media containing 2 mM glutamine (Gibco, 25030-081), N2 supplement (Gibco, 17502-048), B27 supplement (Gibco, 17504-044), and penicillin-streptomycin. The culture media were changed initially after 5 days and then every 3 days. Animals used in the current research were acquired and cared for in accordance with the guidelines published in the National Institutes of Health *Guide for the Care and Use of Laboratory Animals*.

### 2.4 Cytotoxicity Tests

N2a cells (3 × 10^4^ cells/ml) or primary neurons (8 × 10^4^ cells/ml) were grown in 96-well plates and treated with MG132/rotenone or drugs as indicated for 24 h. DMSO was used as a negative control. For measurement of cytotoxicity, Cell Counting Kit-8 (CCK-8; Enzo Life Science, ALX-850-039-KI02) was used according to the manufacturer’s instructions. Briefly, 10 µL of CCK-8 reagent was added to each well, and the plate was incubated at 37°C for 2 h. The absorbance at 450 nm was measured by using a microplate reader (Tecan). Cell viability is expressed as a percentage of the control. All experiments were performed in triplicate.

### 2.5 Flow Cytometric Analysis

N2a (30 × 10^4^ cells/ml) or primary neurons (80 × 10^4^ cells/ml) were detached with trypsin-EDTA and washed twice with cold PBS. The cells were then resuspended in 250 µL of binding buffer (10 mM HEPES, 140 mM NaCl, 2.5 mM CaCl_2_ (pH 7.4)) and incubated with 3 µL of FITC-conjugated Annexin V (apoptotic cells; BD Biosciences) according to the manufacturer’s guide. Then, cells were gently vortexed and incubated for 15 min at room temperature in the dark. After adding Propidium iodide (necrotic cells; 20 μg/ml), flow cytometry was performed within 1 h using MoFlo Astrios (Beckman Coulter).

### 2.6 Quantitative RT-PCR

N2a cells (20 × 10^4^ cells/ml) or primary neurons (40 × 10^4^ cells/ml) were treated with drug for 8 h, and RNA was extracted from cells by using TRIzol plus RNA Purification Kit (Invitrogen, 12183-555) according to the manufacturer’s instructions. cDNA synthesis was performed at 37°C for 120 min with 100 ng of RNA using a High Capacity cDNA Reverse Transcription kit (Applied Biosystems, 4368814). Quantitative RT-PCR was performed using the one-step SYBR^®^ PrimeScript™ RT-PCR kit (Takara Bio Inc, RR420A) according to the manufacturer’s instructions, followed by detection using an Applied Biosystems 7500 Real-Time PCR system (Applied Biosystems). *18S rRNA* was used as an internal control. The 2^−ΔΔCt^ method was used to calculate relative changes in gene expression, as determined by real time PCR experiments (Livak and Schmittgen, 2001).

### 2.7 Immunoblot Analysis

Cells or 20 adult fly heads were homogenized in Cell Lysis Buffer (Cell Signaling Technology, 9803) with protease and phosphatase inhibitor cocktails. The protein concentration of the cell or fly head lysates was determined by a BCA protein assay (Thermo Fisher, 23225). Next, the protein extracts were mixed with 4x Bolt LDS Sample Buffer (Invitrogen) and 10x Bolt Sample Reducing Agent (Invitrogen) and then boiled at 95°C for 5 min. An equal amount of protein from each sample was separated on Bolt 4–12% Bis-Tris gels (Invitrogen, NW04120BOX) or NuPAGE 3–8% Tris-Acetate gels (Invitrogen, EA0378BOX) and transferred to a polyvinylidene difluoride membrane (PVDF; Invitrogen, LC2005). After the membrane was blocked with 5% skim milk in TBS with 0.025% Tween 20, it was probed with antibodies as indicated and detected with an ECL Prime Kit (GE Healthcare, RPN2232). Samples from three independent experiments were used, and the relative expression levels were determined using a Fusion-FX system (Viber Lourmat).

### 2.8 Cyto-ID Autophagy Analysis

N2a cells (3 × 10^4^ cells/ml) or primary neurons (8 × 10^4^ cells/ml) were treated with rapamycin or drugs for 24 h, and the cells were assessed using the Cyto-I Green Autophagy Kit (Enzo Life Science, ENZ-51031-K200) according to the manufacturer’s instructions. Briefly, Cyto-ID dye or Hoechst 33342 was added to each well of a 96-well plate. Then, the plate was incubated at 37°C for 30 min. Cells were washed in 1x Assay Buffer with 2% FBS. The fluorescence signals (excitation/emission 480/530 nm and 340/480 nm) were measured by using a FlexStation 3 Microplate Reader (Molecular Devices). The ratios of the 480/530 signals over the 340/480 signals were calculated for each sample, and the Cyto-ID fluorescence is represented as a percentage of the control. All experiments were performed in triplicate.

### 2.9 Autophagy Assessment

Instead of pellet embedding, flat embedding was used for autophagy assessment in cells (Ylä-Anttila et al., 2009). Without cell harvest, cells on coverslips were fixed at 4°C for 1 h in 2.5% glutaraldehyde and 2% paraformaldehyde in 0.1 M sodium cacodylate buffer (pH 7.4) and postfixed with 2% reduced osmium tetroxide (3% potassium ferrocyanide combined with an equal volume of 4% osmium tetroxide) for 30 min at 4°C. Then, the cells were stained with thiocarbohydrazide (TCH) and 2% osmium tetroxide in distilled water and *en bloc* in 1% uranyl acetate. The cells were then dehydrated via a graded ethanol series and embedded with an EMBed-812 Embedding Kit (EMS). The embedded samples were incubated for 48 h in 60°C. Resin blocks were incubated for 48 h at 60°C. The embedded samples were sectioned (60 nm) with an ultramicrotome (Leica), and the sections were then viewed on a Tecnai 20 transmission electron microscope (TEM; Thermo Fisher) at 120 kV. Images were captured with a US1000X-P camera 200. Stitching images were acquired using Photomontage software (Thermo Fisher). The numbers of autophagosomes and autolysosomes were counted in cells of almost the same size using ImageJ software (National Institutes of Health).

### 2.10 Mitochondrial Activity Assay

For assessment of neuronal mitochondrial dysfunction, N2a cells (4 × 10^4^ cells/ml) and primary neurons (8 × 10^4^ cells/ml) were seeded in XF24-well culture plates (Seahorse Bioscience). The cells were washed twice with XF Base Medium supplemented with 2 mM L-glutamine, 10 mM D-glucose and 1 mM sodium pyruvate (pH 7.4) and incubated at 37°C in a non-CO_2_ incubator for 1 h. Mitochondrial dysfunction was evaluated using the XF Cell Mito Stress Test *Kit* (Seahorse Bioscience) according to the manufacturer’s instructions, followed by measurement using an XF24 Extracellular Flux Analyzer (Seahorse Bioscience). The 24-well utility plate was hydrated, treated with 2 µM oligomycin, 2 μM carbonyl cyanide 4-(trifluoromethoxy) phenylhydrazone (FCCP), and 0.5 µM antimycin A + rotenone, and was then used to calibrate the analyzer. The basal oxygen consumption rate (OCR), ATP production, maximum reserve, and respiratory capacity were calculated as previously described (Dranka et al., 2011), with averages calculated from 4 wells per condition in each individual experiment. The OCR was normalized to the total protein concentration (OD). After the Seahorse analysis, the plate was centrifuged at 280 × *g* for 5 min. The media were aspirated, and the plate was washed twice with PBS. The cells were lysed in RIPA buffer. Protein concentrations in cell lysates were determined using a BCA assay kit.

### 2.11 IncuCyte Live Cell Imaging

N2a cells (2.5 × 10^3^ cells/ml) were plated in 96-well plates and transfected with *Gfp (pCMV-AC-Gfp)* and human *TDP-43 (pCMV-AC-TDP-43-Gfp)* cDNA by using Lipofectamine 3000 reagent (Invitrogen, L3000-015) according to the manufacturer’s instructions. Six hours after transfection, the cells were treated with HEXA-018 (5 µM) and subsequently treated with IncuCyte Red Cytotoxicity Reagent (50 nM; EssenBioscience, 4632). Images were collected with an IncuCyte Zoom System and a ×20 objective lens at 6 h intervals. Cell toxicity was analyzed with IncuCyte ZOOM software by counting the green and red double-positive cells.

### 2.12 Fly Strains

*Drosophila* stocks were maintained on standard cornmeal agar media at 24°C unless otherwise noted. UAS-TARDBP was described previously (Kim et al., 2014). All other stocks were from The Bloomington Stock Center.

### 2.13 Climbing and Lifespan Assays

Adult males (0–1 day old) were separated and transferred into experimental vials containing fly medium mixed with or without RU-486 at a density of 25 (for lifespan) or 25 (for climbing assay) flies per vial (*n* > 100). The number of dead flies was recorded daily, and the flies were transferred to fresh media every other day. Adult locomotor function was assessed by a previously described method (Feany and Bender, 2000), with 100 flies per genotype per time point in all experiments. Experiments were repeated twice to ensure consistent results.

## 3 Results

### 3.1 HEXA-018 Induces Autophagy in an mTOR-independent Manner in Neuronal Cells

**FIGURE 2:**
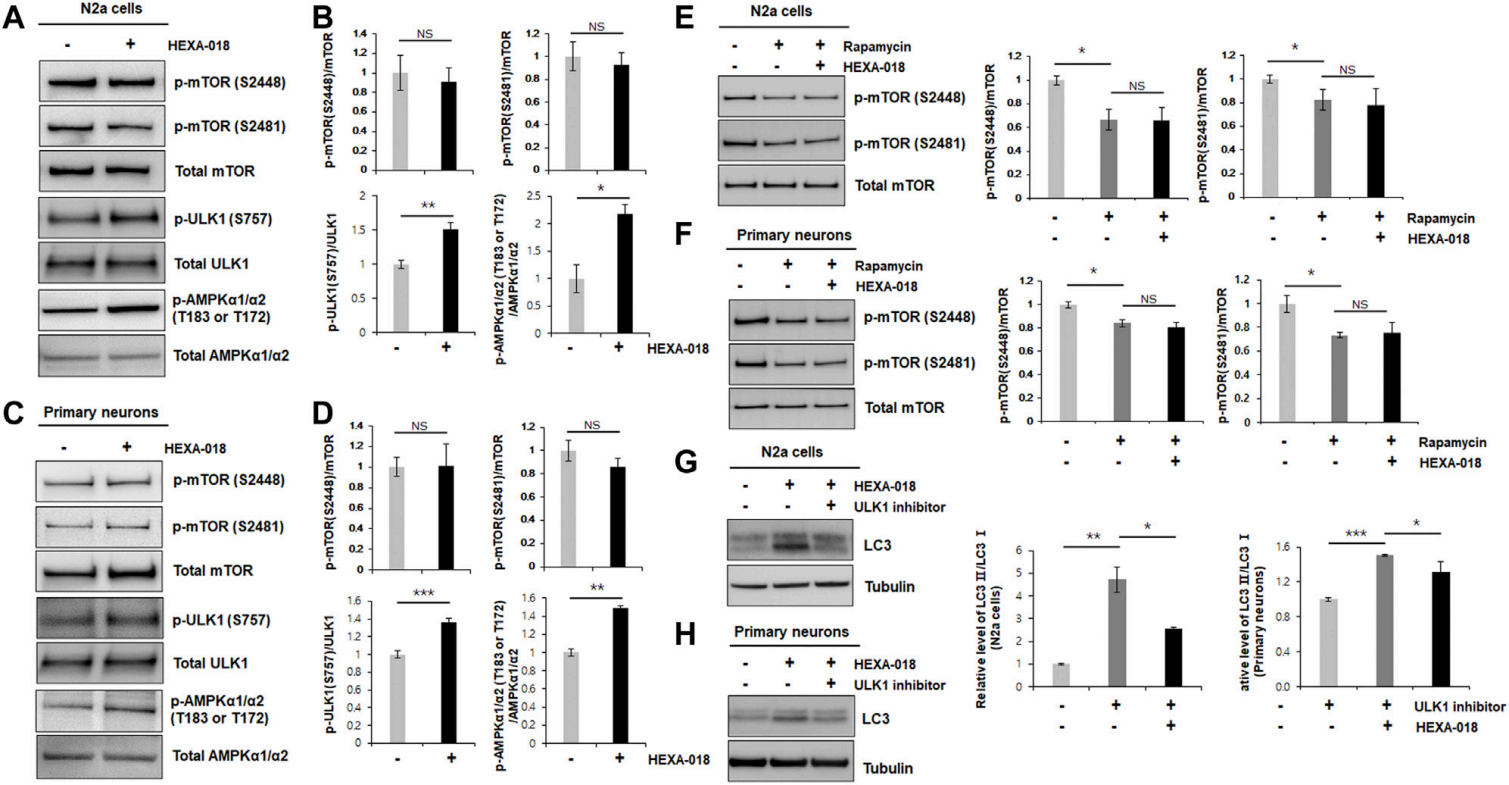
HEXA-018 is an mTOR-independent activator of the ALP. **(A,B)** N2a cells were treated with HEXA-018 (5 µM) for 24 h, and then, the cells were harvested for total protein extraction. Western blot analysis was performed to determine phospho-mTOR (S2481 or S2488), total mTOR, phospho-ULK1 (S757), ULK1, p-AMPKα1/α2 (T183 or T172), or AMPKα1/α2 protein expression. Quantification of the immunoblots was performed from 3 independent experiments. Treatment with HEXA-018 did not affect phospho-mTOR levels but phospho-ULK1 and phospho-AMPK protein levels was significantly increased in N2a cells. *p < 0.05; **p < 0.005; *n. s.*, not significant (unpaired Student’s *t*-test). **(C,D)** Primary neurons were treated with HEXA-018 (0.5 µM) for 24 h, and then, the cells were harvested for total protein extraction. Western blot analysis was performed to determine phospho-mTOR (S2481 or S2488), total mTOR, phospho-ULK1 (S757), ULK1, p-AMPKα1/α2 (T183 or T172), or AMPKα1/α2 protein expression. **p < 0.005; ***p < 0.001; *n. s.*, not significant (unpaired Student’s *t*-test). **(E,F)** N2a cells and primary neurons were pretreated with HEXA-018 (5 µM/0.5 µM) for 30 min and subsequently treated with rapamycin (200 nM) for 24 h, and then, the cells were harvested for total protein extraction. Western blot analysis was performed to determine phospho-mTOR (S2481 or S2488) or total mTOR protein expression. HEXA-018 treatment was not significantly changed following rapamycin-decreased phospho-mTOR levels in N2a cells and primary neurons. Data are presented as the mean ± SD of 3 independent experiments. *p < 0.05; *n. s.*, not significants (one-way ANOVA with Tukey’s multiple comparison test). **(G,H)** N2a cells were pretreated with HEXA-018 (5 µM) for 30 min and subsequently treated with ULK1 inhibitor (200 nM) for 24 h, and then, the cells were harvested for total protein extraction. Western blot analysis was performed to determine LC3-I/II protein expression. HEXA-018 increased LC3-II level was significantly decreased by ULK1 inhibition in N2a cells. Data are presented as the mean ± SD of 3 independent experiments. *p < 0.05; **p < 0.005 (one-way ANOVA with Tukey’s multiple comparison test). **(H)** Primary neurons were pretreated with HEXA-018 (0.5 µM) for 30 min and subsequently treated with ULK1 inhibitor (20 nM) for 24 h, and then, the cells were harvested for total protein extraction. Western blot analysis was performed to determine LC3-I/II protein expression. *p < 0.05; ***p < 0.001 (one-way ANOVA with Tukey’s multiple comparison test).

To investigate whether HEXA-018 activates the ALP, we examined the microtubule-associated protein light chain 3 (LC3-I/II) ratios in N2a cells following treatment with HEXA-018. The increase in the LC3-I/II ratio is a typical indicator of autophagic activation. We found that HEXA-018 treatment significantly increased the LC3-I/II ratio in N2a cells (Figure 1B). Another features of ALP activation are the reduction of p62 protein. However, HEXA-018 did not affect the protein level of p62 in neuronal cells (Figure 1B). We also observed that HEXA-018 treatment upregulated the transcription of *lc3a* and *lc3b* mRNA in N2a cells and primary neurons (Figure 1C). We confirmed these results in primary neurons treated with HEXA-018 (Figures 2D,E). For further confirmation of ALP activation, we used Cyto-ID fluorescence dye. Cyto-ID fluorescence dye specifically labels all types of autophagic vacuoles, including amphisomes or autolysosomes (Guo et al., 2015). Thus, the fluorescence intensities of Cyto-ID-stained cells indicate the level of ALP activation. Rapamycin is a well-known activator of the ALP (Thellung et al., 2019), so the fluorescence intensity of Cyto-ID-stained N2a cells and mouse primary neurons was strongly elevated by rapamycin treatment (Figures 1F,G). Consistent with these results, we observed that the HEXA-018-treated cells showed increased Cyto-ID fluorescence intensities in both N2a cells and primary neurons (Figures 1F,G). These results suggest that HEXA-018 activates the ALP in N2a cells and primary neurons.

The most well-studied regulatory mechanism of the ALP is the mTOR (mechanistic target of rapamycin) pathway. To determine whether HEXA-018 induces autophagy via modulation of the mTOR pathway, we measured the phosphorylation level of mTOR in the HEXA-018-treated N2a cells and primary neurons. The phosphorylation of mTOR (Ser2481 and Ser2448) is critical for mTOR activation. However, the phosphorylation levels of mTOR were not affected by HEXA-018 treatment in N2a cells (Figures 2A,B). We confirmed these results in primary neurons treated with HEXA-018 (Figures 2C,D). These results suggest that HEXA-018 activates the ALP in an mTOR-independent manner. Previous studies demonstrated that the phosphorylation of ULK1 (Ser757) and AMPK1 (Thr183 and Thr172) is the major contributor of ALP activation in mTOR dependent and independent ALP (Jung et al., 2010; Roach, 2011; Din et al., 2012). We analyzed phosphorylation levels of ULK1 and AMPK1 in N2a cells and primary neurons treated with HEXA-018. We showed that HEXA-018 clearly increased the phosphorylation levels of ULK1 and AMPK1 (Figures 2A–D). We used rapamycin as a mTOR-dependent activator of autophagy. We found that the rapamycin treatment significantly decreased the mTOR phosphorylation, but HEXA-018 treatment did not change mTOR phosphorylation in rapamycin-treated neuronal cells (Figures 2E,F). Consistent with previous findings, we observed that rapamycin decreased the level of p62 protein in N2a cells and primary neurons (Supplementary Figures 1A,B). These results suggest that HEXA-018 significantly increased the ULK1 and AMPK1 phosphorylation levels, but mTOR phosphorylation levels were not affected by HEXA-018 treatment. In addition, HEXA-018 induced autophagy activation was significantly decreased by ULK1 inhibition in N2a cells and primary neurons (Figures 2G,H). Taken together, HEXA-018 activates the ALP via the ULK1-AMPK pathway in an mTOR-independent manner.

### 3.2 HEXA-018 Facilitates Autolysosome Formation in Neuronal Cells

**FIGURE 3:**
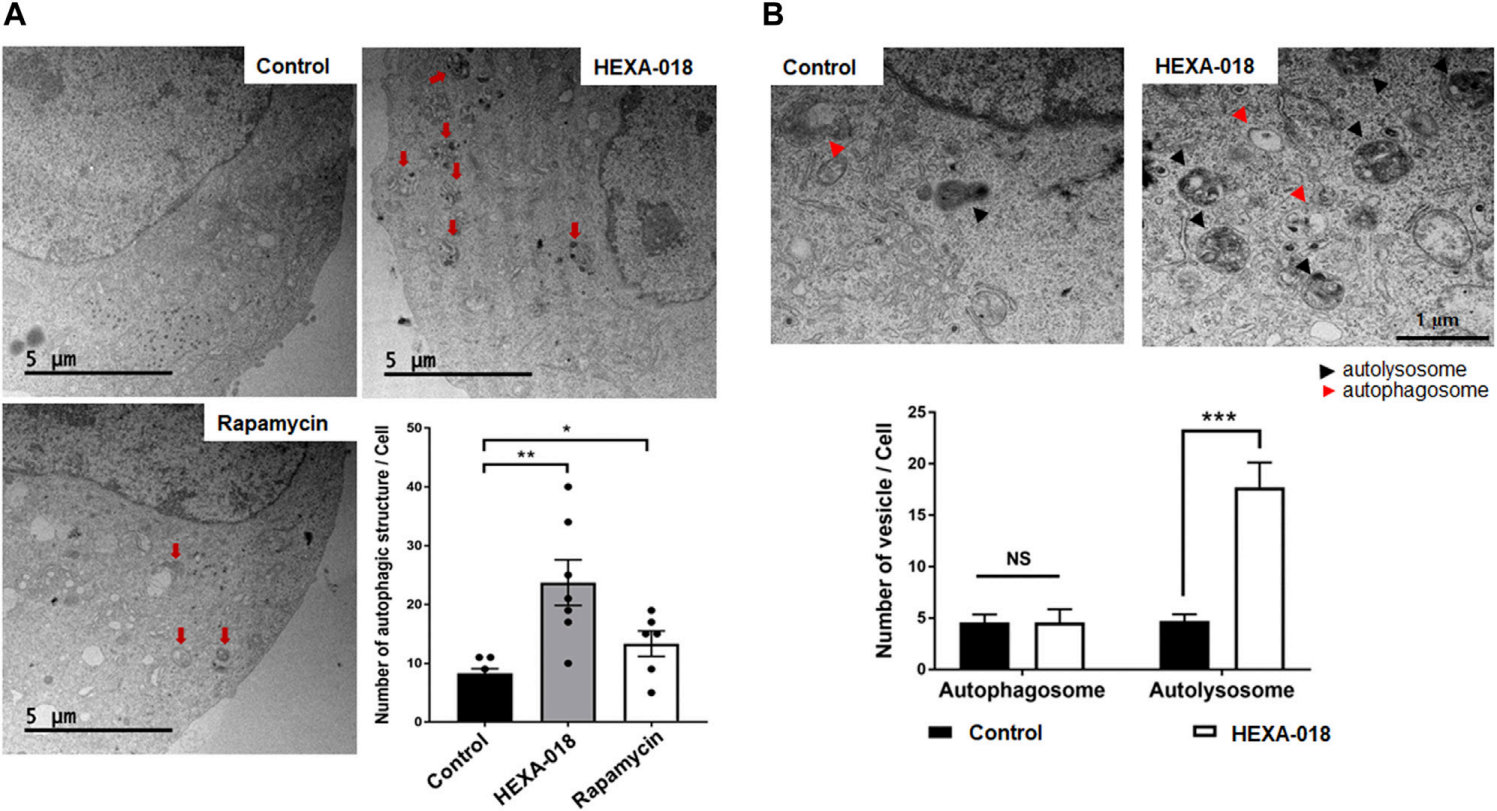
HEXA-018 increases the formation of autolysosomes in neuronal cells. **(A)** Representative TEM images showing autophagy in N2a cells and Hexa-018- or rapamycin-treated cells. N2a cells were treated with HEXA-018 (5 µM) for 24 h and then analyzed. Rapamycin (200 nM) was used as a positive control. Electron microscopy images show that the number of autophagic structures and vesicles was significantly increased by HEXA-018 in N2a cells. The red arrows indicate autophagic structures. Quantification of the number of autophagic structures and vesicles per cell. *p < 0.05; **p < 0.005 (one-way ANOVA with Tukey’s multiple comparison test). **(B)** The arrowheads indicate autophagosomes (red) and autolysosomes (black). Quantification of the number of autophagosomes and autolysosomes from the images (*n* = 7) is shown in the graph. The bar graph shows the mean ± SEM of the representative groups. ***p < 0.001; *n. s.*, not significant (one-way ANOVA with Tukey’s multiple comparison test).

Electron microscopy is one of the most precise ways to detect and quantify autophagic structures. To investigate whether HEXA-018 increases the number of autophagic vacuoles, we analyzed autophagic structures using transmission electron microscopy (TEM). With autophagy initiation, the phagophore fully surrounds its cargo and fuses to form the double-membrane autophagosome. After fusion with the lysosome, strong electron density represented as the criterion for identification of autolysosomes due to degradation of materials. Various autophagic structures, including autophagosomes and autolysosomes, were observed in N2a cells. The red arrows in Figure 3A indicate autophagic structures. The number of autophagic vacuoles was substantially increased after HEXA-018 or rapamycin treatment in N2a cells (Figure 3A). To investigate the regulatory role of HEXA-018 in autolysosome formation, we fluorescent puncta assay with DsRed-LC3-GFP, which is a well-established method to monitor autophagy flux (Sheen et al., 2011). This assay uses the characteristics that the GFP fluorescence is lost but DsRed fluorescence is stable in the lysosomal acidic environment. The autophagic vesicles, both autophagosome (yellow puncta) and autolysosome (red puncta), were significantly increased by HEXA-018 and rapamycin treatment in N2a cells (Supplementary Figure S2). We then further assessed the numbers of autophagosomes and autolysosomes in the control and HEXA-018-treated cells using TEM (Figure 3B). The number of autophagosomes was not significantly changed, but the number of autolysosomes was significantly increased in the HEXA-018-treated cells compared with the control cells (Figure 3B). These results strongly suggest that HEXA-018 activates the ALP. Considering that autolysosome formation was significantly increased by HEXA-018 treatment, HEXA-018 appears to promote the fusion of autophagosomes and lysosomes.

### 3.3 HEXA-018 Ameliorates UPS Impairment and ROS-Induced Neurotoxicity

**FIGURE 4:**
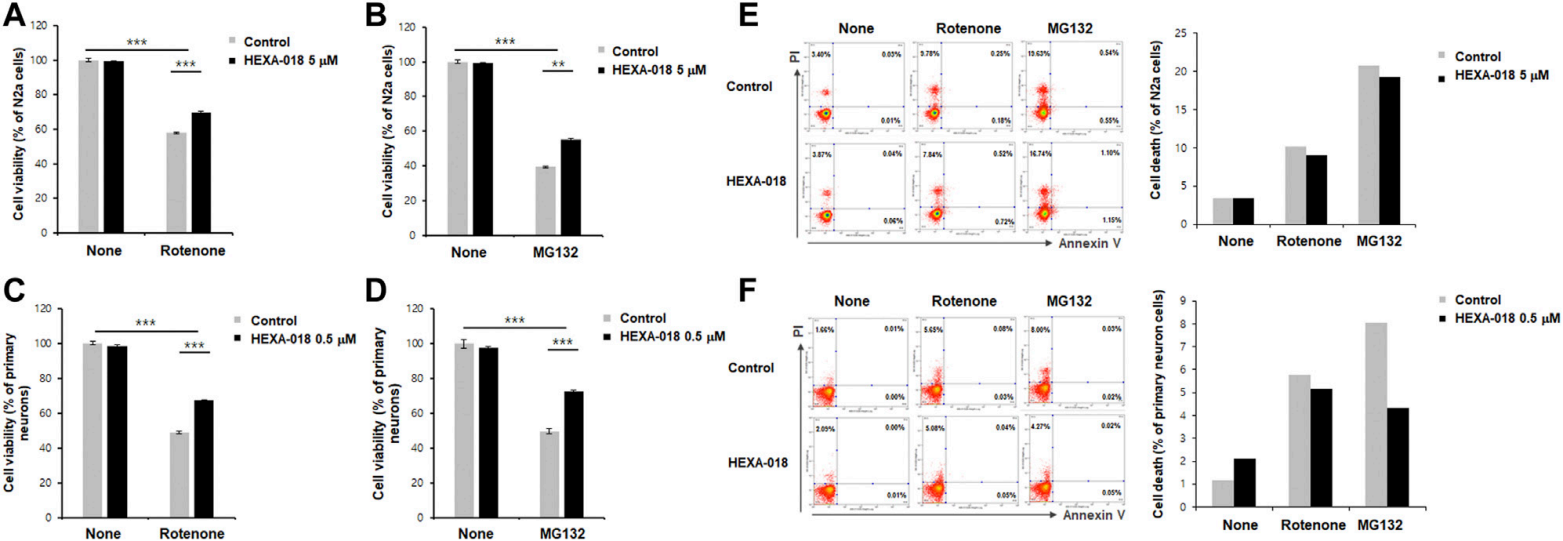
HEXA-018 attenuates MG132-and rotenone-induced toxicity in neuronal cells. **(A,B)** N2a cells were pretreated with HEXA-018 (5 µM) for 30 min and subsequently treated with MG132 (5 μM, **(A)**) or rotenone (10 μM, **(B)**) for 24 h. CCK-8 analysis was performed thereafter. HEXA-018 significantly reduced MG132-or rotenone-induced neuronal toxicity in N2a cells. Data are presented as the mean ± SD of 3 independent experiments. **p < 0.005; ***p < 0.001 (one-way ANOVA with Tukey’s multiple comparison test). **(C,D)** Primary neurons were pretreated with HEXA-018 (0.5 µM) for 30 min and subsequently treated with MG132 (5 μM, **(C)**) or rotenone (10 μM, **(D)**) for 24 h. MG132-or rotenone-induced neuronal toxicity was also reduced by HEXA-018 treatment in primary neurons. CCK-8 analysis was performed thereafter. 5 µM or 0.5 µM were the maximum non-cytotoxic concentrations of HEXA-018 in N2a cells and primary neurons, respectively. HEXA-018 concentration was then assessed after 24 h treatment using the each highest concentrations showing no toxicity in N2a cells and primary neurons. Data are presented as the mean ± SD of 3 independent experiments. ***p < 0.001 (one-way ANOVA with Tukey’s multiple comparison test). **(E,F)** N2a cells and primary neurons were pretreated with HEXA-018 (5/0.5 µM) for 30 min and subsequently treated with MG132 (5 µM) or rotenone (10 µM) for 24 h. FACS analysis was additional performed to assess cell viability after HEXA-018 treatment against neurotoxic drugs (10,000 cells per each condition). Representative scatter plots show that annexin V^−^/PI^+^ (upper left quadrant, necrosis) and annexin V^+^/PI^+^ (upper right quadrant, late apoptosis/necrosis) were significantly increased by MG132 or rotenone, but annexin V^+^/PI^−^ (lower right quadrant, early apoptosis) was not significantly changed in N2a cells and primary neurons. The proportion of cells residing in each quadrant is represented as a percentage. The percentage of cell death (apoptosis and necrosis) in the flow cytometric analysis is shown **(*right*).**

MG132 (proteasome inhibitor) and rotenone (inducer of reactive oxygen species (ROS)) are currently accepted as neurotoxicity-inducing factors. Previous studies have shown that ALP activation mitigates MG132-or rotenone-induced neurotoxicity. Thus, we investigated the protective effect of HEXA-018 in MG132-or rotenone-treated N2a cells and primary neurons. HEXA-018 significantly attenuated the cytotoxicity of MG132 and rotenone in N2a cells (Figures 4A,B; Supplementary Figures 3A,B) and primary neurons (Figures 4C,D; Supplementary Figures 3C,D). We also confirmed these results using a different experimental approach based on flow cytometry using Annexin V and PI staining. We found that HEXA-018 attenuated the rotenone/MG132-induced necrotic cell death in N2a (Figure 4E; Supplementary Figure 4A) and primary neurons (Figure 4F; Supplementary Figure 4B). These results indicate that HEXA-018 mitigates UPS impairment and ROS-induced neurotoxicity.

**FIGURE 5:**
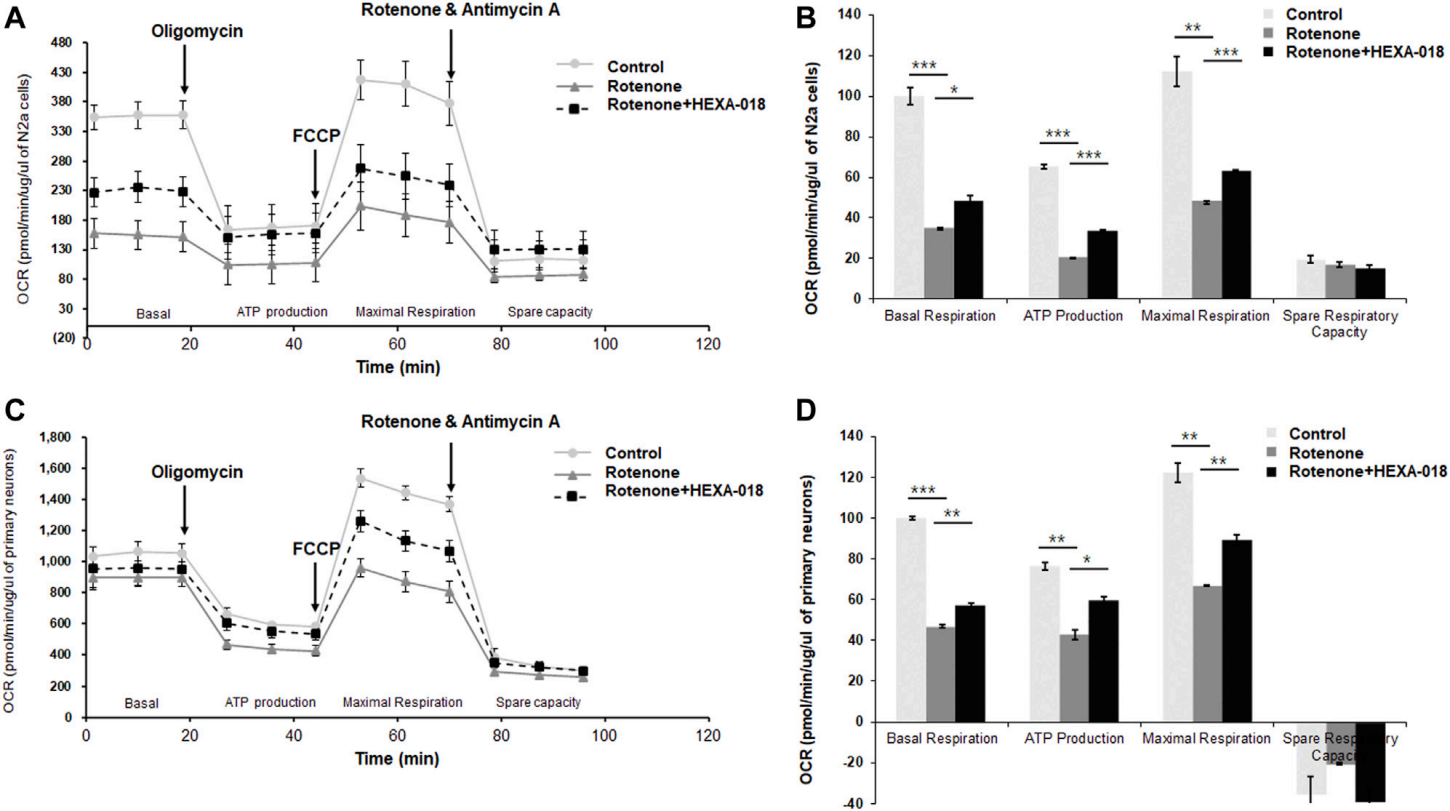
HEXA-018 mitigates rotenone-induced mitochondrial dysfunction in neuronal cells. **(A,B)** N2a cells were pretreated with HEXA-018 (5 µM) for 30 min and subsequently treated with rotenone (10 μM) for 24 h and then analyzed. **(A)** Mitochondrial function was analyzed in N2a cells by detecting the basal OCR, ATP production, maximum reserve, and respiratory capacity with a Seahorse XF analyzer. The OCR was normalized to the total protein concentration. **(B)** Quantification of the OCR, ATP production, maximum reserve, and respiratory capacity as percentages of the basal values. Data are presented as the mean ± SD. *p < 0.05; **p < 0.005; ***p < 0.001 (two-way ANOVA with Tukey’s multiple comparison test). **(C,D)** Primary neurons were pretreated with HEXA-018 (0.5 µM) for 30 min and subsequently treated with rotenone (10 μM) for 24 h and then analyzed. **(C)** Mitochondrial function was analyzed in primary neurons by detecting the basal OCR, ATP production, maximum reserve, and respiratory capacity with a Seahorse XF analyzer. The OCR was normalized to the total protein concentration. **(D)** Quantification of the OCR, ATP production, maximum reserve, and respiratory capacity as percentages of the basal values. Data are presented as the mean ± SD. *p < 0.05; **p < 0.005; ***p < 0.001 (two-way ANOVA with Tukey’s multiple comparison test).

Recent studies have suggested that overproduction of ROS leads to mitochondrial damage and that mitochondrial dysfunction is a key pathological feature of many neurodegenerative diseases, such as ALS, AD, and PD. Accumulating evidence suggests that autophagy activation suppresses rotenone-induced neurotoxicity such as mitochondrial dysfunction and oxidative stress in cell and mice (Chen et al., 2007; Lin et al., 2012; Xiong et al., 2013). Moreover, rotenone-induced α-synuclein aggregates are significantly decreased by rapamycin treatment (Yu et al., 2009). Thus, we next investigated whether HEXA-018 suppresses rotenone-induced mitochondrial dysfunction in neuronal cells. N2a cells and primary neurons were treated with HEXA-018 and rotenone, and then, we measured the cellular oxygen consumption rate (OCR) using the Seahorse XF24 Extracellular Flux Analyzer and a mitochondrial stress test kit. OCR is an indicator of mitochondrial respiration, including basal respiration, ATP production, maximal respiration, and spare respiratory capacity. Analysis of mitochondrial respiratory parameters was performed by using oligomycin, FCCP, and antimycin A + rotenone. Notably, we found that the basal respiration, ATP production, and maximal respiratory parameters were markedly decreased in the rotenone-treated cells compared to the control cells. The rotenone-induced reductions in basal respiration, ATP production, and maximal respiration parameters were strongly ameliorated by HEXA-018 treatment, but the spare respiratory capacity was not altered (Figures 5A–D). Taken together, these findings suggest that HEXA-018 attenuates rotenone-induced mitochondrial dysfunction in neuronal cells.

### 3.4 HEXA-018 Suppresses TDP-43-Induced Toxicity in Mammalian Cells and Flies

**FIGURE 6:**
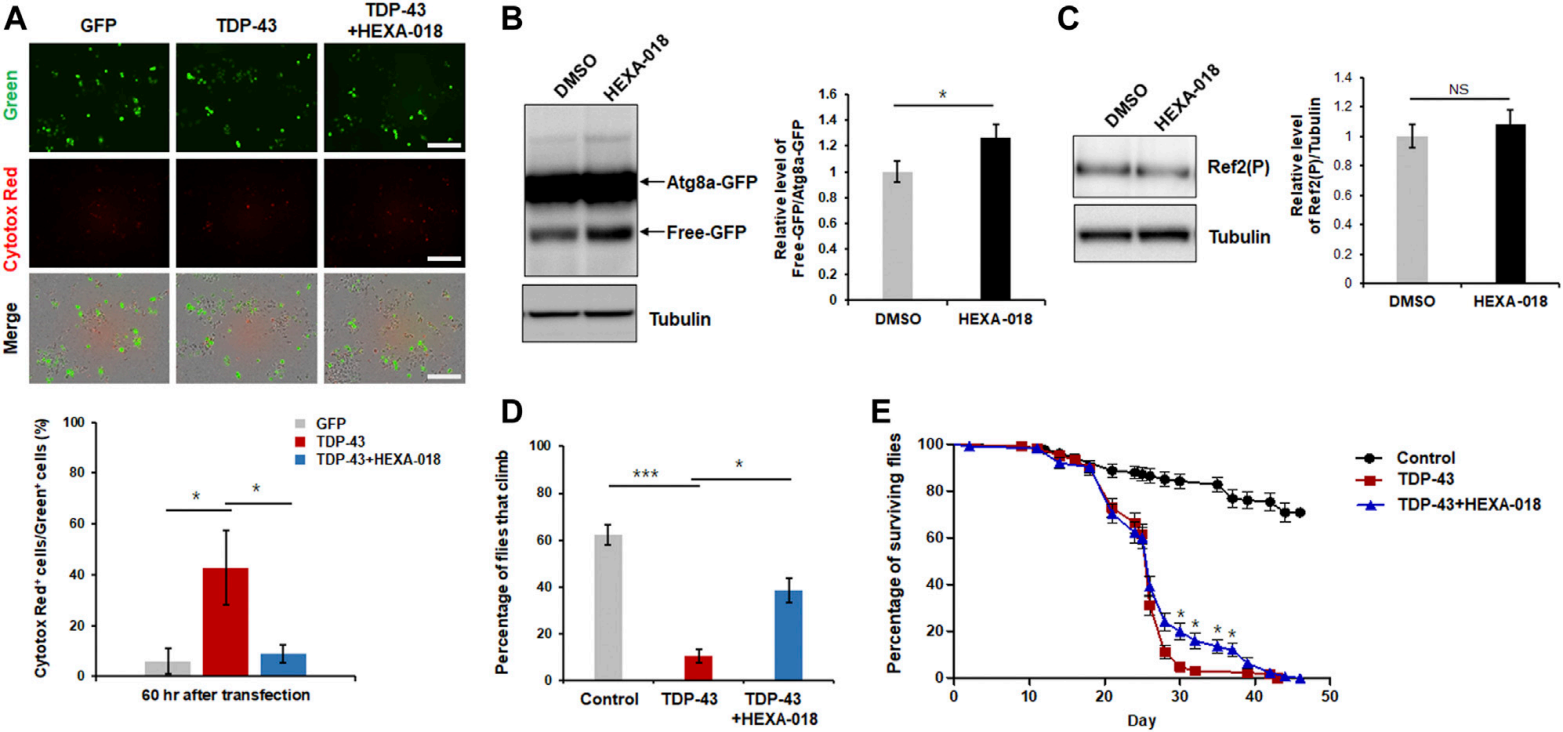
HEXA-018 suppresses TDP-43-induced toxicity in neuronal cells and *Drosophila*. **(A)** N2a cells were transfected with the GFP or TDP-43-GFP expression construct for 6 h and subsequently treated with HEXA-018 (5 µM) for 54 h. Dying neuronal cells, as indicated by red fluorescence, were imaged every 3 h and quantified by counting double-fluorescent (green and red) cells using the IncuCyte system. HEXA-018 significantly reduced TDP-43-induced neuronal toxicity. Data are presented as the mean ± SD of 3 independent experiments. *p < 0.05 (one-way ANOVA with Tukey’s multiple comparison test). Scale bar, 200 μm. **(B)** HEXA-018 treatment increased ALP activation. Western blot analysis of Atg8a-GFP and free GFP protein levels in the head lysates of flies. Quantification of immunoblots was performed from 3 independent experiments, and protein levels were normalized to that of tubulin. Data are presented as the mean ± SD. Genotypes: *elavGS, UAS-Atg8a-GFP/+*. *p < 0.05 (unpaired Student’s *t*-test). **(C)** Western blot analysis of Ref2(P) protein levels in the head lysates of Atg8a-GFP flies. Quantification of immunoblots was performed from 3 independent experiments, and protein levels were normalized to that of tubulin. HEXA-018 treatment did not affect Ref2(P) protein level. Data are presented as the mean ± SD. Genotypes: *elavGS, UAS-Atg8a-GFP/+*. *n. s.*, not significant (unpaired Student’s *t*-test). **(D,E)** Climbing ability and lifespan of TDP-43 flies fed HEXA-018 (10 μM) at the indicated time points. The TDP43-induced motility deficit was significantly reduced by treatment with HEXA-018. The percentages of flies that climbed or survived were quantified. Genotypes: control is *elavGS/+*, TDP-43 is e*lavGS, UAS-TARDBP/+.* Data are presented as the mean ± SEM (*n* = 4 replicates per condition, each with *n* = 25 flies per sample). *p < 0.05; ***p < 0.001 (one-way ANOVA with Tukey’s multiple comparison test).

Previous studies have indicated that overexpression of TDP-43 cause neuronal toxicity in mammalian and *Drosophila* neurons. In addition, oxidative stress and UPS impairment are key pathological features in TDP-43 proteinopathy. All these data suggest that HEXA-018 might attenuate TDP-43-induced neurotoxicity. Thus, we analyzed TDP-43 toxicity using a live cell imaging system (IncuCyte). GFP- or GFP-tagged TDP-43-expressing N2a cells were treated with HEXA-018, and the cell death of the GFP-expressing cells was monitored. As expected, TDP-43-induced neuronal toxicity was significantly suppressed by HEXA-018 treatment (Figure 6A).

We also assessed ALP activity in a *Drosophila in vivo* model. To evaluate ALP function, we expressed the Atg8a-GFP reporter in the fly nervous system. The ratio of free GFP to Atg8a-GFP correlates with ALP activity because globular GFP protein is much more resistant to acidic lysosomal conditions than Atg8a. By adopting this method, we examined the free GFP/Atg8a-GFP ratio in the fly model. We found that the ratio was significantly higher in the HEXA-018-treated flies than in the control flies (Figure 6B). Moreover, we found that Ref2(P) (*Drosophila* homologue of p62) protein level was not changed in Atg8a-GFP flies (Figure 6C). These data suggest that HEXA-018 activates the ALP in fly neurons. Given the strong *in vitro* evidence that HEXA-018 attenuates TDP-43-induced toxicity in neuronal cells, we next examined whether HEXA-018 treatment suppresses TDP-43-induced toxicity in a *Drosophila* model of TDP-43 proteinopathy that expresses human TDP-43 in the nervous system (Kim et al., 2014; Lee et al., 2020a). We also found that HEXA-018 treatment significantly improved the TDP-43-induced climbing deficit and shortened lifespan compared with the control treatment (Figures 6D,E). Taken together, these results show that HEXA-018 ameliorates TDP-43 toxicity in fly and mammalian cell line models of TDP-43 proteinopathy via ALP activation.

## 4 Discussion

In this study, we demonstrated the effect of HEXA-018, a novel compound containing a catechol derivative structure, as an inducer of autophagy. Recent studies have shown that the ALP has many roles, including cellular homeostasis, metabolism, development, antitumor properties, and innate defense (Levine and Kroemer, 2008; Mizushima and Levine, 2010; Mizushima and Komatsu, 2011; Wang and Levine, 2011; Kim and Lee, 2014). Moreover, dysregulation of the ALP is closely associated with neurodegenerative diseases, such as Alzheimer’s disease, amyotrophic lateral sclerosis (ALS), and Parkinson’s disease (Ravikumar et al., 2004; Crews et al., 2010; Spilman et al., 2010; Barmada et al., 2014; Hsueh et al., 2016). Thus, ALP activation is a promising therapeutic strategy for neurodegenerative diseases.

The mTOR signaling pathway is the most well-characterized negative regulator of the ALP (Dunlop and Tee, 2014). In addition, several studies have shown that rapamycin has beneficial effects in some animal models of age-associated neurodegenerative diseases via ALP activation (Jahrling and Laberge, 2015). However, mTOR activation in the brain plays essential roles in synapse development, neurotrophic factor synthesis, neuronal apoptosis and neuroinflammation (Kim, 2014; Jahrling and Laberge, 2015; Kim et al., 2018; Kim, 2020). Furthermore, human clinical studies have suggested that rapamycin induces various side effects, including immunosuppression, impaired wound healing, hyperlipidemia, and proteinuria (Stallone et al., 2009; Salmon, 2015). Thus, developing mTOR-independent inducers of autophagy could be a promising therapeutic strategy for neurodegenerative diseases.

HEXA-018 is a novel compound with a catechol derivative structure. Previous studies have demonstrated that urushiol, a catechol derivative, induces autophagic flux (Go et al., 2017). Moreover, catechol derivatives mediate autophagy-mediated cell death in cancer cells (Hong et al., 1999; Kim et al., 2013; Go et al., 2017). In this study, we found that HEXA-018 activates the ALP in an mTOR-independent manner. HEXA-018 specifically increased the number of autolysosomes (Figure 3B). This result suggests that HEXA-018 facilitates the fusion of autophagosomes with lysosomes. The studied regulators of autophagosome-lysosome fusion are Ras-associated binding (Rab) GTPases, including Rab2 and Rab7 (Lorincz and Juhasz, 2020). However, the soluble N-ethylmaleimide–sensitive factor attachment protein receptor (SNARE) complex and vesicular transport system are also essential for the fusion process. Therefore, further studies are warranted to determine how HEXA-018 modulates the ALP. The possible mTOR-independent ALP activation pathways of HEXA-018 are Ca2+, inositol and IP3, ULK1/AMPK, and MAPK/JNK pathway (Williams et al., 2008; Decuypere et al., 2011; Kim et al., 2011; Zhao et al., 2013; Li et al., 2015). To elucidate the mechanism of HEXA-018 mediated ALP activation, we performed additional experiment to test whether HEXA-018 is related to the ULK1/AMPK pathway. We found that the HEXA-018 activates the ULK1/AMPK pathway. Moreover, ULK1 inhibition is sufficient to suppress HEXA-018-induced ALP activation. However, there is still a possibility that HEXA-018 could increase ALP activation via multiple pathways.

As for the concentration of HEXA-018, we have treated with several concentrations of HEXA-018 in primary neurons and N2a cells and then selected one concentration (for each cell types) that does not exhibit cytotoxicity itself and have significant protective effect against neurotoxic agents. Many studies have found significant differences between immortalized cell lines and primary neurons. Indeed, immortalized cells or those derived from tumors differ biologically from normal, differentiated neurons obtained from the fetal brain. Notably, HEXA-018 alone showed a dose dependent response with high doses (N2a cells; 100 µM and primary neurons; 1 µM) demonstrating significant toxicity while lower doses (N2a cells; 1, 5, 10 µM and primary neurons; 0.1, 0.5 µM) had no effect on cell toxicity using FACS analysis (Supplementary Figure S3). Moreover, treatment with rotenone and MG132 significantly increased the necrotic rates, but apoptotic rate were not affected in N2a cells and primary neurons by flow cytometry. The treatment with HEXA-018 also decreased the rotenone-induced necrotic rate (Figures 4E,F).

TDP-43 is the major component of inclusions or aggregates present in the neuronal cells of patients affected by ALS and frontotemporal lobal degeneration (FTLD) (Liscic et al., 2008; Scotter et al., 2015; James et al., 2016; Josephs et al., 2017). Importantly, TDP-43-induced UPS impairment plays a critical role in the pathogenesis of TDP-43 by regulating neurotoxicity. Moreover, ALP activation reduces TDP-43 aggregation, cytoplasmic mislocalization and toxicity in mouse and cultured neurons (Ayala et al., 2011; Avendano-Vazquez et al., 2012; Wang et al., 2012; Barmada et al., 2014). Taken together, all evidence suggests that regulation of ALP plays an important role in the TDP-43 proteinopathy model. We demonstrated that HEXA-018 significantly attenuated the ALP activation and behavioral defect induced by TDP-43-expressing cells and flies.

Our data suggest that HEXA-018 suppressed neuronal toxicity in cell and *Drosophila* models of TDP-43 proteinopathy. These results present the possibility that HEXA-018-mediated ALP activation may be a novel therapeutic intervention for neurodegenerative diseases with TDP-43 proteinopathy.

## Data Availability

The original contributions presented in the study are included in the article/Supplementary Material, further inquiries can be directed to the corresponding authors.
