# Supplementary material for: HEXA-018, a Novel Inducer of Autophagy, Rescues TDP-43 Toxicity in Neuronal Cells
Source: Front Pharmacol. 2021 Dec 2;12:747975. doi: 10.3389/fphar.2021.747975 (PMC8675103; doi:10.3389/fphar.2021.747975)
Supplement: Supplementary file 1 [file DataSheet1.pdf]

1 Supplementary Methods

1.1 Autophagy assessment

For monitoring autophagosome formation or autophagic flux, N2a cells (6×10^4 cells/ml) on coverslips were plated and transiently transfected with DsRed-GFP-LC3 using Lipofectamine 3000 reagent (Invitrogen, L3000-015) according to the manufacturer's instructions. After 48 h of transfection, cells were treated with rapamycin or HEXA-018 for 4 h, and then cells were fixed in 4% paraformaldehyde (PFA) in PBS for 30 min. The samples were mounted with 4′,6-diamidino-2-phenylindole (DAPI) and observed with a confocal microscope (Leica) Photomicrographs from three randomly chosen 20 fields were captured, and the number of autophagosome and autolysosome was counted in cells.

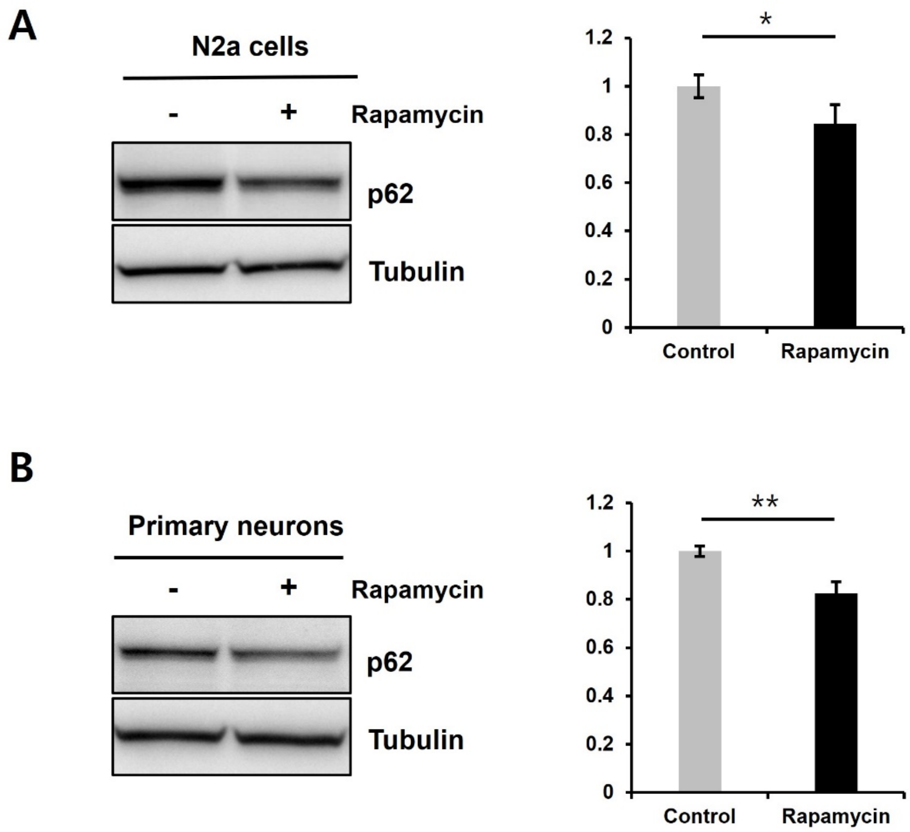

Supplementary Figure S1. Rapamycin decreases the level of p62 protein in neuronal cells. N2a cells and primary neurons were treated with rapamycin (200 nM) for 24 h, and then, the cells were harvested for total protein extraction. Western blot analysis was performed to determine p62 protein expression. Data are presented as the mean ± SD of 3 independent experiments. *$p<0.05$; **$p<0.005$ (unpaired Student's *t*-test).

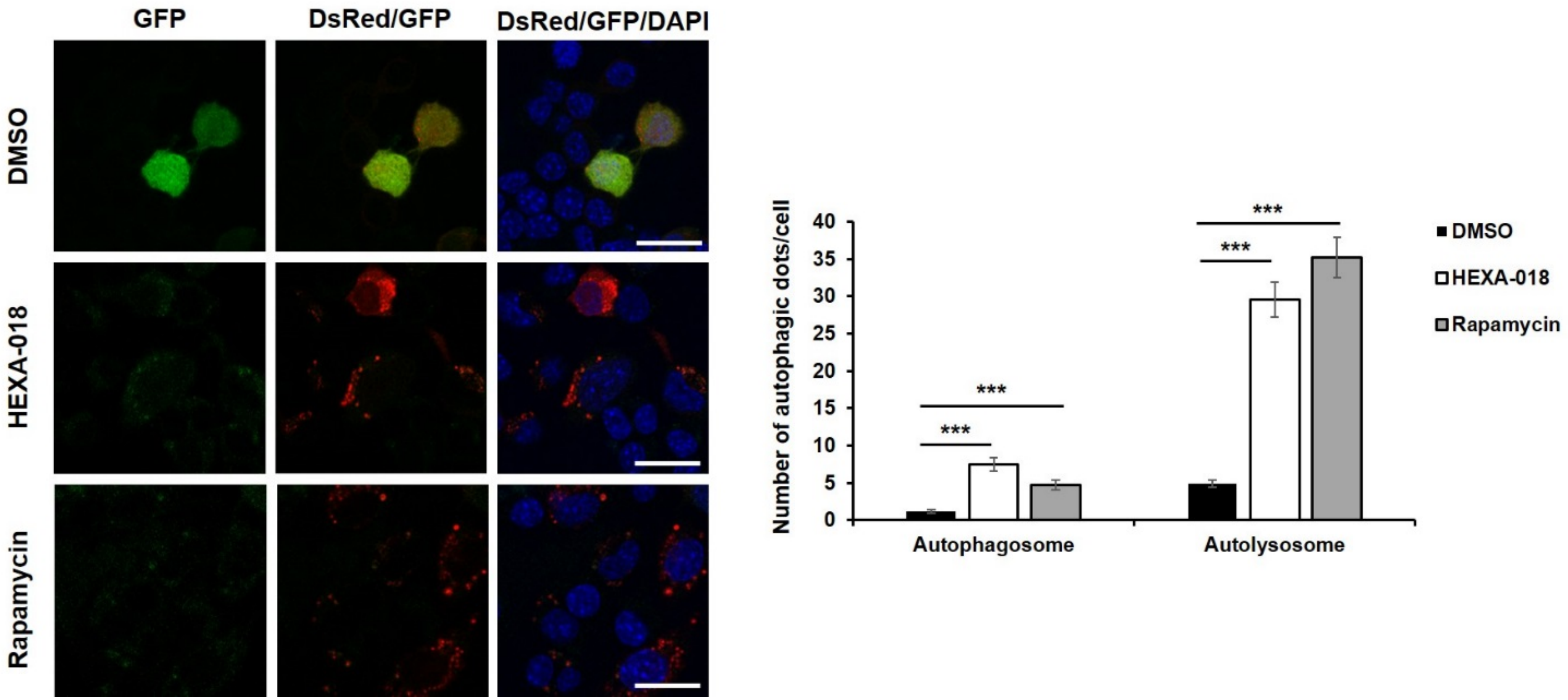

Supplementary Figure S2. HEXA-018 increases the formation of autophagic vesicle in neuronal cells. N2a cells were transfected with the DsRed-LC3-GFP expressing construct for 48 h and subsequently treated with HEXA-018 (5 µM) or Rapamycin (200 nM) for 4 h and then cells were fixed in 4% paraformaldehyde (PFA). Confocal microscopy images show that the number of both autophagosome and autolysosome was significantly increased by HEXA-018. Images show representative fields with autophagosomes (yellow) and autolysosomes (red). Quantification of yellow and red puncta in individual cells was performed by manual counting. Scale bar, 25 µm. Twenty cells were examined in each groups. Data are presented as the mean ± SEM. ***p<0.001 (one-way ANOVA with Tukey's multiple comparison test).

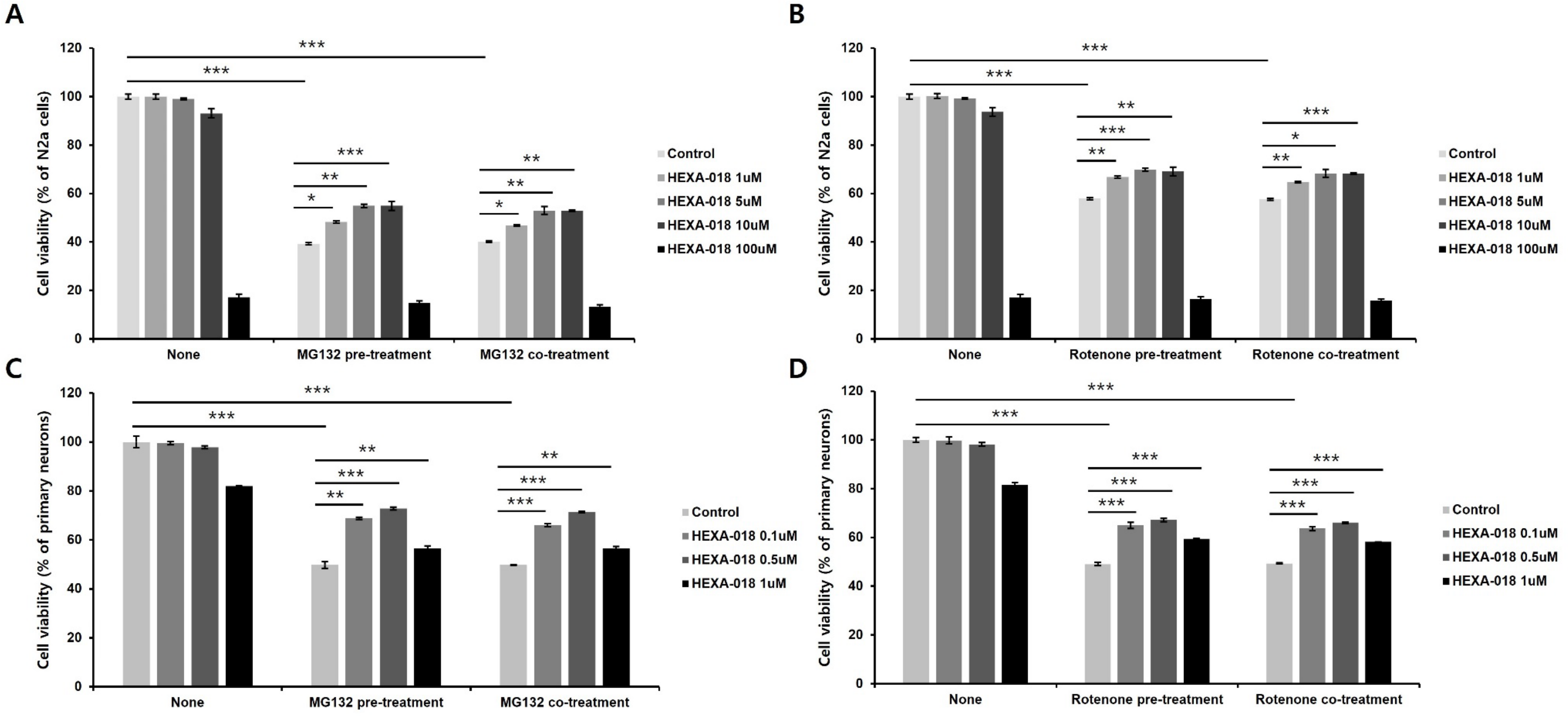

Supplementary Figure S3. The effects of pre-treatment and co-treatment of HEXA-018 in various concentrations on MG132- or rotenone-induced neuronal cell toxicity. (A-B) In pre-treatment condition, N2a cells were pretreated with HEXA-018 (1, 5, 10, or 100 μM) and subsequently treated with MG132 (5 μM, **A**) or rotenone (10 μM, **B**) for 24 h. In co-treatment condition, N2a cells were cotreated with HEXA-018 and MG132 (or rotenone). CCK-8 analysis was performed thereafter. Data are presented as the mean ± SD of 3 independent experiments. **p*<0.05; ***p*<0.005; ****p*<0.001 (one-way ANOVA with Tukey's multiple comparison test). **(C-D)** In pre-treatment condition, primary neurons were pretreated with HEXA-018 (0.1, 0.5, or 1 μM) for 30 min and subsequently treated with MG132 (5 μM, C) or rotenone (10 μM, D) for 24 h. In co-treatment condition, N2a cells were cotreated with HEXA-018 and MG132 (or rotenone).

CCK-8 analysis was performed thereafter. Data are presented as the mean ± SD of 3 independent experiments. **p<0.005; ***p<0.001 (one-way ANOVA with Tukey's multiple comparison test).

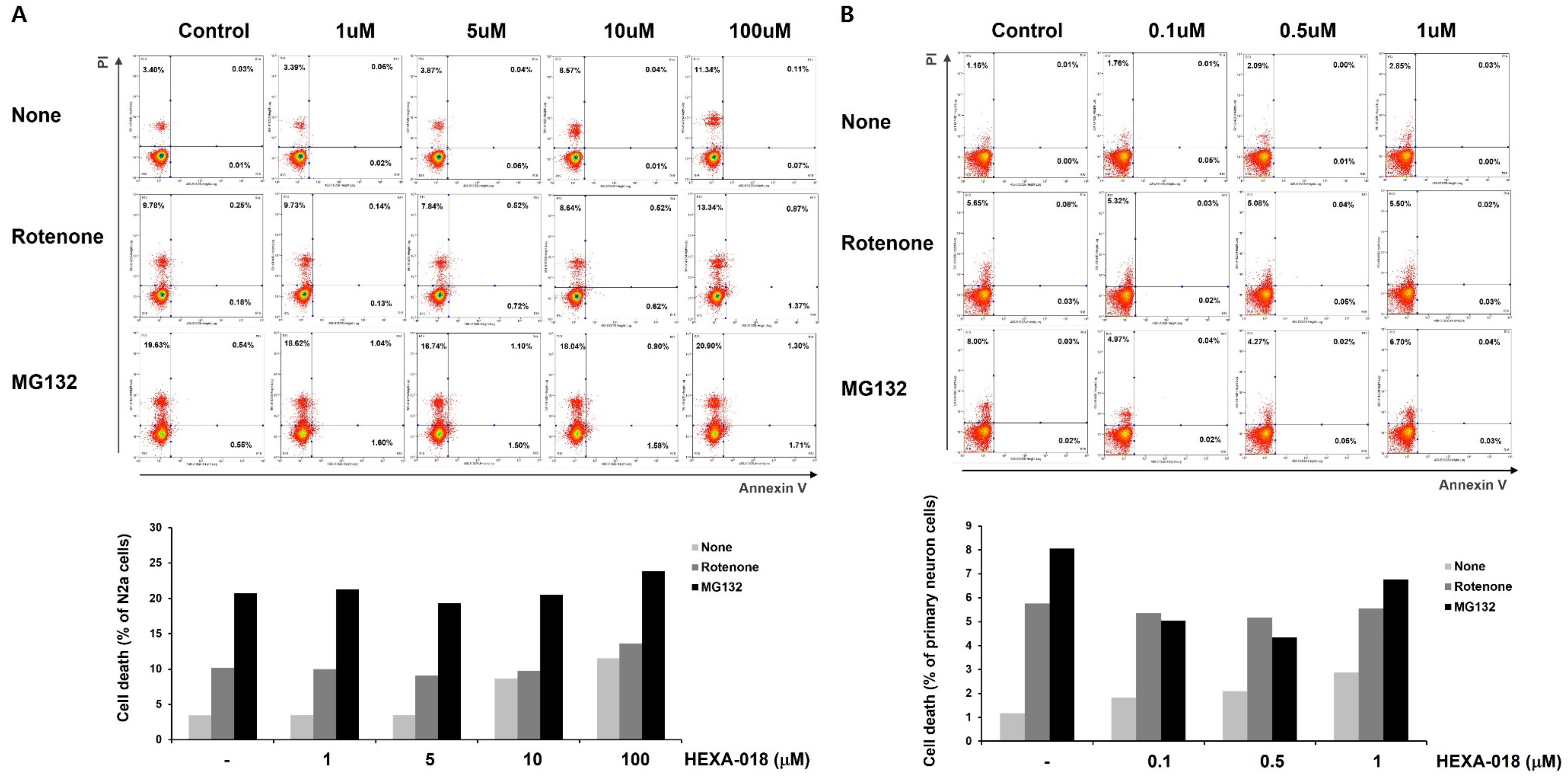

Supplementary Figure S4. The effects of HEXA-018 in various concentrations on MG132- or rotenone-induced neurotoxicity using flow cytometry analysis. (A) N2a cells were pretreated with HEXA-018 (1, 5, 10, or 100 μM) for 30 min and subsequently treated with MG132 (5 μM) or rotenone (10 μM) for 24 h. Flow cytometric analysis was performed using Annexin V and PI staining (10,000 cells per each condition). Representative scatter plots show that annexin V^-/PI^+ (upper left quadrant, necrosis) and annexin V^+/PI^+ (upper right quadrant, late apoptosis/necrosis) were significantly increased by MG132 or rotenone, but annexin V^+/PI^- (lower right quadrant, early apoptosis) was not significantly changed in N2a cells and primary neurons. The proportion of cells residing in each quadrant is represented

as a percentage. The percentage of cell death (apoptosis and necrosis) in the flow cytometric analysis is shown (*below*). **(B)** Primary neurons were pretreated with HEXA-018 (0.1, 0.5, or 1 μM) for 30 min and subsequently treated with MG132 (5 μM) or rotenone (10 μM) for 24 h. Flow cytometric analysis was performed using Annexin V and PI staining. The percentage of cell death (apoptosis and necrosis) in the flow cytometric analysis is shown (*below*).
